# A systematic review and meta-analysis of active case finding for tuberculosis in India

**DOI:** 10.1016/j.lansea.2022.100076

**Published:** 2022-09-17

**Authors:** Tushar Garg, Lelia H. Chaisson, Fahd Naufal, Adrienne E. Shapiro, Jonathan E. Golub

**Affiliations:** a Department of Epidemiology, Johns Hopkins Bloomberg School of Public Health, Baltimore, MD, United States; b Division of Infectious Diseases, Department of Medicine, University of Illinois at Chicago, Chicago, IL, United States; c Wilmer Eye Institute, Johns Hopkins Medicine, Baltimore, MD, United States; d Department of Global Health and Department of Medicine, University of Washington, Seattle, WA, United States; e Department of Medicine, Johns Hopkins School of Medicine, Baltimore, MD, United States; f Department of International Health, Johns Hopkins Bloomberg School of Public Health, Baltimore, MD, United States

**Keywords:** Tuberculosis, Active case finding, ACF, Screening, Number needed to screen, India

## Abstract

**Background:**

Active case finding (ACF) for tuberculosis (TB) is the cornerstone case-finding strategy in India's national TB policy. However, ACF strategies are highly diverse and pose implementation challenges in routine programming. We reviewed the literature to characterise ACF in India; assess the yield of ACF for different risk groups, screening locations, and screening criteria; and estimate losses to follow-up (LTFU) in screening and diagnosis.

**Methods:**

We searched PubMed, EMBASE, Scopus, and the Cochrane library to identify studies with ACF for TB in India from November 2010 to December 2020. We calculated 1) weighted mean number needed to screen (NNS) stratified by risk group, screening location, and screening strategy; and 2) the proportion of screening and pre-diagnostic LTFU. We assessed risk of bias using the AXIS tool for cross-sectional studies.

**Findings:**

Of 27,416 abstracts screened, we included 45 studies conducted in India. Most studies were from southern and western India and aimed to diagnose pulmonary TB at the primary health level in the public sector after screening. There was considerable heterogeneity in risk groups screened and ACF methodology across studies. Of the 17 risk groups identified, the lowest weighted mean NNS was seen in people with HIV (21, range 3–89, *n*=5), tribal populations (50, range 40–286, *n*=3), household contacts of people with TB (50, range 3-undefined, *n*=12), people with diabetes (65, range 21-undefined, *n*=3), and rural populations (131, range 23–737, *n*=5). ACF at facility-based screening (60, range 3-undefined, *n*=19) had lower weighted mean NNS than at other screening locations. Using the WHO symptom screen (135, 3-undefined, *n*=20) had lower weighted mean NNS than using criteria of abnormal chest x-ray or any symptom. Median screening and pre-diagnosis loss-to-follow-up was 6% (IQR 4.1%, 11.3%, range 0–32.5%, *n*=12) and 9.5% (IQR 2.4%, 34.4%, range 0–86.9%, *n*=27), respectively.

**Interpretation:**

For ACF to be impactful in India, its design must be based on contextual understanding. The narrow evidence base available currently is insufficient for effectively targeting ACF programming in a large and diverse country. Achieving case-finding targets in India requires evidence-based ACF implementation.

**Funding:**

WHO Global TB Programme.

**Research in context**

**Evidence before this study**

The World Health Organization (WHO) has provided recommendations for systematic screening for tuberculosis in 2021. This review was part of a larger systematic review of the active case-finding (ACF) across multiple populations and risk groups that informed the guideline development.

**Added value of this study**

Our study provides translational evidence for implementing ACF in India. The number needed to screen (NNS) for identifying one case of TB was low for people with HIV, tribal people, household contacts of people with TB, and people with diabetes. ACF via facility-based screening had lower NNS than at other screening locations. The WHO symptom screen had lower weighted mean NNS than using chest x-ray or any symptom. Pre-diagnosis loss to follow-up was high, and likely to impact the yield of ACF programs. There was high variability in ACF implementation in India and sparse literature for many important risk groups.

**Implications of all the available evidence**

We recommend the Indian National TB Elimination Program prioritize ACF in groups with a high risk of TB and address losses to follow-up in the ACF care cascade. We also highlight the need for additional evidence for effective ACF strategies in India.

Alt-text: Unlabelled box

## Introduction

Globally, India has the highest burden of tuberculosis (TB), with an estimated 2.59 million new cases and 504,000 deaths in 2020.^1^ Despite a significant increase in TB case notifications in India in recent years, half a million incident cases still go undetected and unnotified, and India continues to fall short of targets for closing the case-finding gap. This inadequate progress in increasing case notifications has only been exacerbated by the disruption in TB care resulting from the COVID-19 pandemic.^2^^,^^3^ In 2020, India achieved approximately three-fourths of the TB notification target set at the 2018 UN High Level Meeting on Tuberculosis, while registering a 25% relative decline in notifications compared to 2019.

Early identification of people with active TB and administering prompt treatment is a cornerstone of case finding that disrupts TB transmission in communities, attenuates health and financial impacts of TB, and improves access to TB care.^4^ Reaching the half a million “missing” cases requires an alternative to passive case-finding, which requires that people with TB present to a health facility for diagnosis. Active case-finding (ACF) comprises a collection of health provider-initiated approaches that involves actively screening people for TB and initiating treatment for those diagnosed.^5^ The underlying principle is to screen high-risk populations who have a high risk of TB and an unmet need for TB services.^6^ These approaches have varying complexity in their implementation, use of screening tools, and selection of target populations.

India's National TB Elimination Program (NTEP) characterizes ACF as the lynchpin of its case-finding strategy in the National Strategic Plan for 2017–2025 (NSP).^7^^,^^8^ The NSP advocates a multisectoral approach for case detection that includes “vulnerability mapping, systematic screening of high-risk groups, and systematic screening for TB symptoms in health care institutions.”^8^ The NSP suggests ACF should be prioritized in 28 vulnerable groups, while simultaneously highlighting its poor yield and resource intensiveness. The NTEP currently operationalizes ACF in selected high-risk groups using facility-based healthcare workers and mobile vans. In 2020 and 2021, this strategy had a yield of one diagnosed case for 3290 and 305 people screened, respectively.^3^^,^^9^ The large differences in yield reflects the need for optimization in terms of populations targeted, screening and diagnostic tools, and operational challenges. The Joint Monitoring Mission in 2019 raised similar concerns, highlighting the need for formative evaluations that can inform ACF programming, including target population, high-yield screening location, and screening criteria.^10^ Therefore, we conducted a systematic review of the literature to summarize evidence from India and determine the number needed to screen (NNS) to identify one case of TB across different risk groups, strategies, and screening criteria.

## Methods

### Research objective

Our primary objectives were to determine the NNS to identify one case of TB across various risk groups, screening locations, and screening criteria as a summary statistic for ACF implementations in India. Our secondary objective was to estimate losses to follow-up before screening and diagnosis in the ACF cascade.

### Search strategy and selection criteria

This review was part of a larger systematic review of the NNS for ACF across multiple populations and risk groups that served as an update of the 2013 review by Shapiro et al.^11^, ^12^, ^13^ and was conducted on behalf of the WHO in order to inform a Guideline Development Group (GDG) meeting to update the recommendations on systematic screening for TB.^14^ We searched four databases (PubMed, EMBASE, Scopus, and the Cochrane library) for articles on ACF using a combination of terms like “tuberculosis”, “mass screening”, “contact tracing”, and “screening” in our search strategy (Supplementary file 1). We used the original search strategy from November 2010 to February 2020, and then a modified version to update the search with studies from India through December 2020. We imported all abstracts into a Covidence database and removed duplicates. For the initial review, two reviewers independently screened titles and abstracts and disagreements were resolved by consensus or a third reviewer. We included all original research published in English, French, or Spanish which indicated use of ACF.

For the full-text screening, two reviewers independently assessed articles for eligibility and any disagreement was resolved by consensus or a third reviewer. We included original research studies that reported results of ACF for TB. We excluded studies if: 1) Xpert or TB culture was not performed to confirm active TB (for study populations with adults >15 years); 2) the number of persons screened was not reported; 3) outcomes of active and passive case finding were not disaggregated; 4) microbiologic and clinical diagnoses of TB were not disaggregated (for study populations with adults >15 years); 5) only an abstract was published; 6) the paper was not in English, French, or Spanish; 7) the paper presented duplicate data from another publication; or 8) the paper was unavailable. For this analysis, we also excluded articles that did not report results from India.

Using a standardized process, one reviewer extracted data related to study characteristics and results from the included papers. We extracted study location, demographic information of the study population, screening location, health sector and level of care, screening and diagnostic criteria, number eligible for screening, number screened, number eligible for testing, number tested, and number of TB cases detected. We extracted data on each risk group separately where multiple risk groups were described. We used a modified version of the AXIS (Appraisal tool for Cross-Sectional Studies) tool for cross-sectional studies to assess risk of bias.^15^

**Figure 1:**
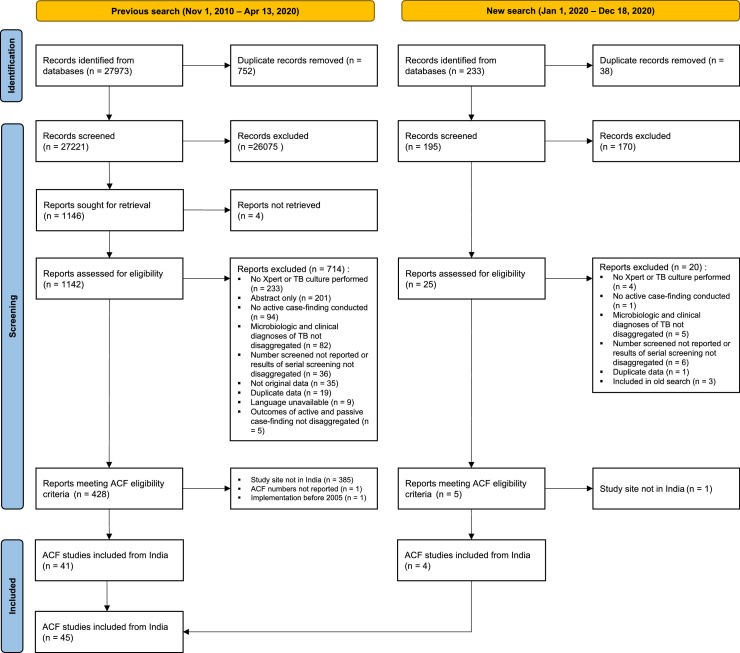
PRISMA diagram. Abbreviations TB, Tuberculosis; ACF, active case finding.

We used the Preferred Reporting Items for Systematic Reviews and Meta-analyses (PRISMA) reporting guidelines (completed checklist available in supplementary file 2).^16^ While study screening included two separate processes, we extracted data from all the included papers at once (PRISMA diagram in Figure 1). We didn't prepare a protocol for this review.

### Definitions

Our *study population* was the number of people eligible or registered for screening as per the individual study definition. We used the number of people screened according to the individual study criteria for the NNS calculations. We defined *active TB* as a microbiological diagnosis of TB using mycobacterial culture or Xpert MTB/RIF for people >15 years. In studies reporting both Xpert and culture results, we defined active TB as culture-positive TB. For children ≤15 years, we defined active TB as any diagnosis of TB disease according to the individual study criteria, including clinical diagnoses. We defined *primary screening* as criteria applied for screening the full study population. For example, a primary screen of WHO symptom screen means that everyone was screened by asking or assessing the presence of symptoms, and a positive screen would then prompt a diagnostic test. We used individual study definitions for extracting the number of people eligible or registered for screening, number of people eligible for testing, and number tested. We defined *screening loss to follow-up* as people eligible or registered for screening who do not undergo screening and *pre-diagnosis loss to follow-up* as people eligible for diagnostic testing (i.e., who had a positive TB screen as per original study criteria) who do not receive testing.

### Analysis

Our primary outcome was the NNS to detect one case of active TB. We calculated this as the inverse of yield from screening for each study, which is proportion with active TB detected in the study population. In addition to the individual NNS for each study, we calculated weighted mean NNS with ranges to account for substantial differences in study population sizes. We calculated weighted mean NNS as the inverse of weighted mean of yield in each study using number of people screened for weights. A study that detected no active TB cases had zero yield and an undefined NNS but contributed to weighted NNS estimates as these were generated after calculating the weighted yield. All NNS values were rounded up to the nearest integer value.

Our secondary outcomes were screening and pre-diagnosis loss to follow-up. We included studies that reported the number of people eligible or registered for screening and number of people screened, and/or the number of people eligible for testing and number tested. For screening loss to follow-up, we included studies with the primary screening criteria of any symptom. Further, we excluded studies using facility-based screening strategies since screening loss to follow-up is null in such studies by design. For pre-diagnostic loss to follow-up, we included studies where diagnostic criteria was culture or Xpert. We calculated proportion lost to follow-up for individual studies, and reported medians, interquartile ranges, and ranges.

We used descriptive statistics to summarize study characteristics, including geography, target population, type of TB diagnosed, and level of health system. We stratified analyses based on risk groups, screening locations, and primary screening criteria. Studies that included multiple risk groups were included in multiple weighted NNS calculations. If studies reported results of serial screening in the same population, we used results from the first round of screening for the weighted NNS calculations, to reflect the yield of screening in a previously unscreened population. Since few studies used secondary screening, we did not stratify based on this criterion. We used R version 4.0.4 and Tableau 2021.1 for analysis.

### Role of funding source

The funders had no role in study design, data collection, data analysis, interpretation, or writing of the report.

## Results

Of the 28,206 records identified from four databases, we screened 27,416 titles and abstracts after removing 790 duplicates. We reviewed 1167 full-texts, and identified 45 eligible studies from India that reported data for 46 ACF interventions conducted after 2005 (one study reported screening for two different populations,^17^ Figure 1).

### Study characteristics

**Figure 2:**
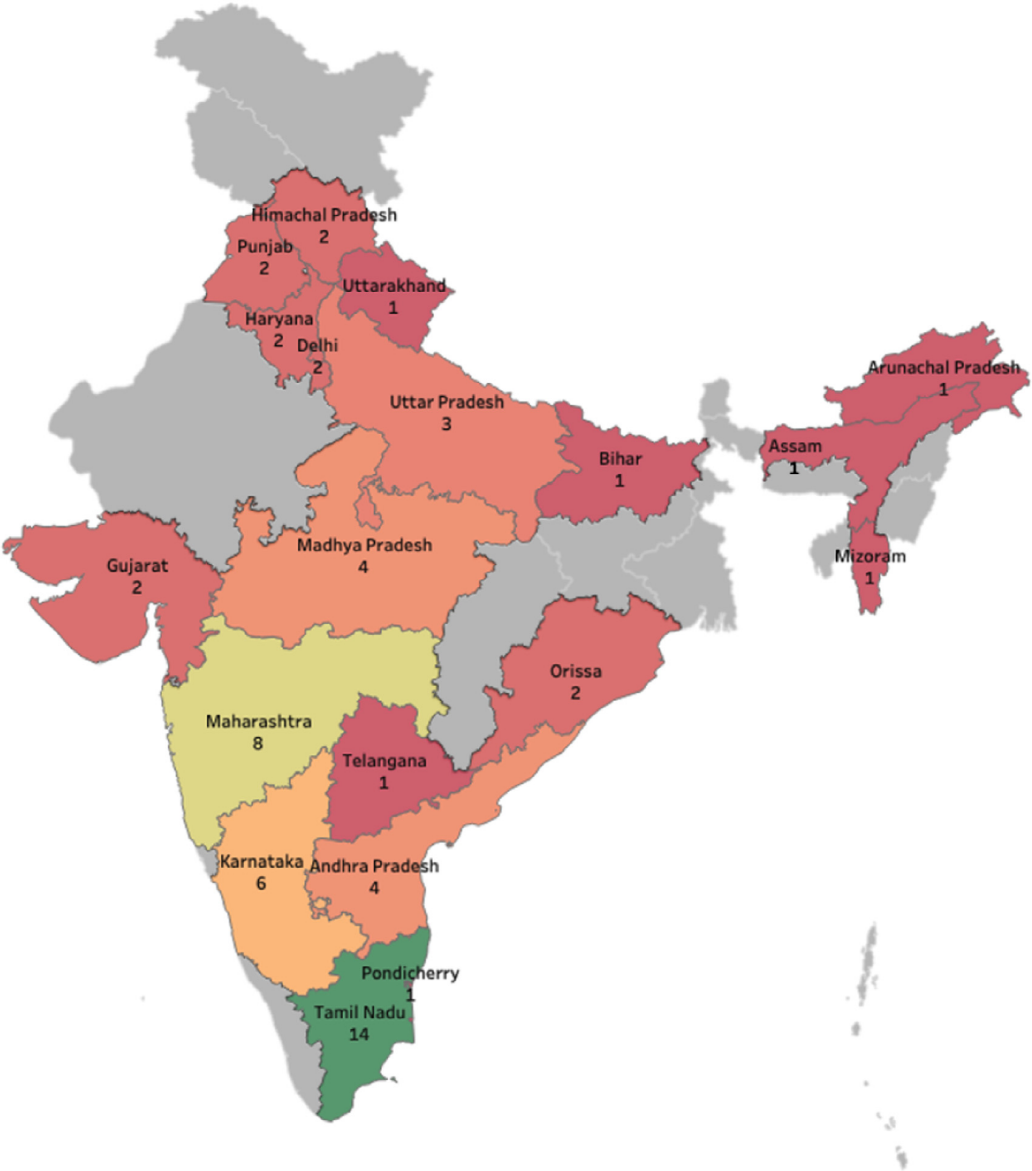
Geographical distribution of active case finding implementations in India. Figure 2 represents 44 studies with implementations in 58 study sites. Where a single study was conducted in multiple states, each instance was counted separately for the geographical characteristic. Further, multiple extractions from a single study were counted only once.^17^ Study in Padmapriyadarshini, 2016 didn't specify the names of four states it was implemented in; therefore, it is not represented in this map.^22^

The studies were conducted in 19 of the total 36 states and union territories of India. (Figure 2) Of the 45 studies, 6 were conducted in multiple states.^18^, ^19^, ^20^, ^21^, ^22^, ^23^ One study conducted in 4 states did not report the location details.^22^ Amongst 58 study sites in 44 studies, 26 were in South India (14 in Tamil Nadu,^18^, ^19^, ^20^, ^21^^,^^24^, ^25^, ^26^, ^27^, ^28^, ^29^, ^30^, ^31^, ^32^, ^33^ 6 in Karnataka,^18^^,^^19^^,^^23^^,^^34^, ^35^, ^36^ 4 in Andhra Pradesh,^20^^,^^37^, ^38^, ^39^ 1 in Telangana,^40^ and 1 in Pondicherry^41^), and 10 in Western India (8 in Maharashtra,^18^^,^^19^^,^^42^, ^43^, ^44^, ^45^, ^46^, ^47^ 2 in Gujarat^18^^,^^19^).

Of the 45 studies, 40 were conducted in a public sector setting,^17^, ^18^, ^19^, ^20^, ^21^, ^22^, ^23^, ^24^, ^25^, ^26^, ^27^, ^28^, ^29^, ^30^^,^^33^, ^34^, ^35^, ^36^, ^37^, ^38^, ^39^, ^40^, ^41^^,^^43^, ^44^, ^45^^,^^47^, ^48^, ^49^, ^50^, ^51^, ^52^, ^53^, ^54^, ^55^, ^56^, ^57^, ^58^, ^59^, ^60^ 3 in private sector,^32^^,^^42^^,^^61^ and 2 were public-private mix.^31^^,^^46^ Ten studies were in urban populations,^25^^,^^29^, ^30^, ^31^^,^^33^^,^^41^^,^^42^^,^^46^^,^^55^^,^^58^ 9 in rural,^21^^,^^24^^,^^26^^,^^27^^,^^36^^,^^48^^,^^49^^,^^53^^,^^59^ 21 in both rural and urban populations^18^, ^19^, ^20^^,^^22^^,^^23^^,^^32^^,^^34^^,^^35^^,^^38^^,^^43^, ^44^, ^45^^,^^47^^,^^50^, ^51^, ^52^^,^^54^^,^^56^^,^ ^57^^,^^60^^,^^61^; the composition was unclear in 5 studies.^17^^,^^28^^,^^37^^,^^39^^,^^40^ The level of health system for TB care varied: 23 at primary level,^17^^,^^21^^,^^24^, ^25^, ^26^, ^27^^,^^29^, ^30^, ^31^^,^^33^^,^^35^^,^^36^^,^ ^38^^,^^39^^,^^48^^,^^49^^,^^51^^,^^53^, ^54^, ^55^^,^^58^, ^59^, ^60^ 2 at secondary level,^20^^,^^50^ 12 at tertiary level,^32^^,^^40^, ^41^, ^42^, ^43^, ^44^, ^45^^,^^47^^,^^52^^,^^56^^,^^57^^,^^61^ 5 had a combination of the three levels,^18^^,^^19^^,^^23^^,^^34^^,^^46^ and 3 studies did not specify.^22^^,^^28^^,^^37^ A total of 33 studies diagnosed only pulmonary TB,^18^, ^19^, ^20^, ^21^^,^^24^, ^25^, ^26^, ^27^, ^28^, ^29^, ^30^, ^31^^,^^34^, ^35^, ^36^, ^37^, ^38^, ^39^, ^40^^,^^43^, ^44^, ^45^^,^^47^, ^48^, ^49^, ^50^, ^51^^,^^53^, ^54^, ^55^^,^^57^^,^^58^^,^^60^ and 12 diagnosed both pulmonary and extra-pulmonary TB.^17^^,^^22^^,^^23^^,^^32^^,^ ^33^^,^^41^^,^^42^^,^^46^^,^^52^^,^^56^^,^^59^^,^^61^ (Table 1, Supplementary File 3)

**Table 1:** Characteristics of study included in the review (*n*=45).

Characteristics	Overall (*n*=45)
**Health sector**	
Private	3 (6.7%)
Public	40 (88.9%)
Public-Private mix	2 (4.4%)
**Population type**	
Mixed	21 (46.7%)
Rural	9 (20.0%)
Unclear	5 (11.1%)
Urban	10 (22.2%)
**Health system level**	
Mixed	5 (11.1%)
Primary	23 (51.1%)
Secondary	2 (4.4%)
Tertiary	12 (26.7%)
Unclear	3 (6.7%)
**Type of TB**	
PTB and EPTB	12 (26.7%)
PTB only	33 (73.3%)

Abbreviations

PTB, Pulmonary TB; EPTB, Extrapulmonary TB

### Number needed to screen

The NNS ranged from 2 to 4203. Three studies had an undefined NNS because no confirmed TB cases were identified.^39^^,^^44^^,^^51^ The crude NNS estimates for individual studies are reported in supplementary file 3.

### Risk group

**Figure 3:**
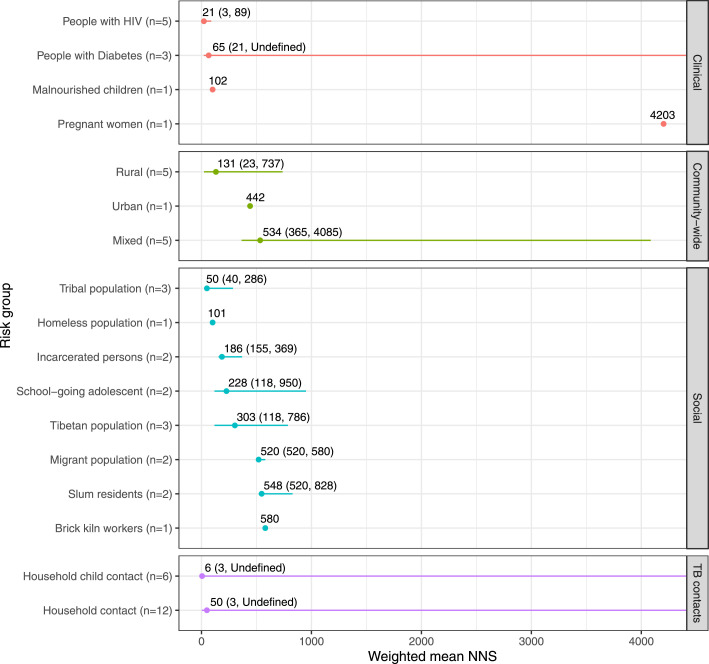
Weighted mean number needed to screen stratified by risk groups. Weighted mean NNS (range) Abbreviations NNS, number needed to screen.

The weighted mean NNS estimates for risk groups are presented in Figure 3.

### Clinical risk groups

The weighted mean NNS was 21 (range 3–89, *n*=5) in people with HIV^22^^,^^42^^,^^45^, ^46^, ^47^ and 65 (21-undefined, *n*=3) in people with diabetes.^32^^,^^40^^,^^44^ The NNS was 102 (*n*=1) in malnourished children^34^ and 4203 (*n*=1) in pregnant women.^41^

### Community risk groups

In ACF targeting entire communities, the weighted mean NNS was 131 (23–737, *n*=5) in rural communities,^21^^,^^26^^,^^27^^,^^36^^,^^59^ 442 (*n*=1) in urban communities,^25^ and 534 (365–4085, *n*=5) in studies with both urban and rural communities.^18^^,^^19^^,^^43^^,^^54^^,^^60^

### Social risk groups

The weighted mean NNS was 50 (40–286, *n*=3) in tribal population,^48^^,^^49^^,^^53^ 101 (*n*=1) in people experiencing homelessness,^29^ 186 (155–369, *n*=2) in prison inmates,^30^^,^^50^ 228 (118–950, *n*=2) in school-going adolescents,^17^^,^^38^ 303 (118–786, *n*=3) in Tibetan refugee populations.^17^^,^^23^ 520 (520–580, *n*=2) in migrant population,^24^^,^^58^ 548 (520–828, *n*=2) in slum residents,^55^^,^^58^ 580 (*n*=1) in brick kiln workers.^24^

### Contact of people with TB

The weighted mean NNS was six (3-undefined, *n*=6) in household child contacts of people with TB^35^^,^^39^^,^^51^^,^^52^^,^^56^^,^^61^ and 50 (3-undefined, *n*=12) in all household contacts of people with TB.^20^^,^^28^^,^^31^^,^^33^^,^^35^^,^^37^^,^^39^^,^^51^^,^^52^^,^^56^^,^^57^^,^^61^

### Screening location

**Figure 4:**
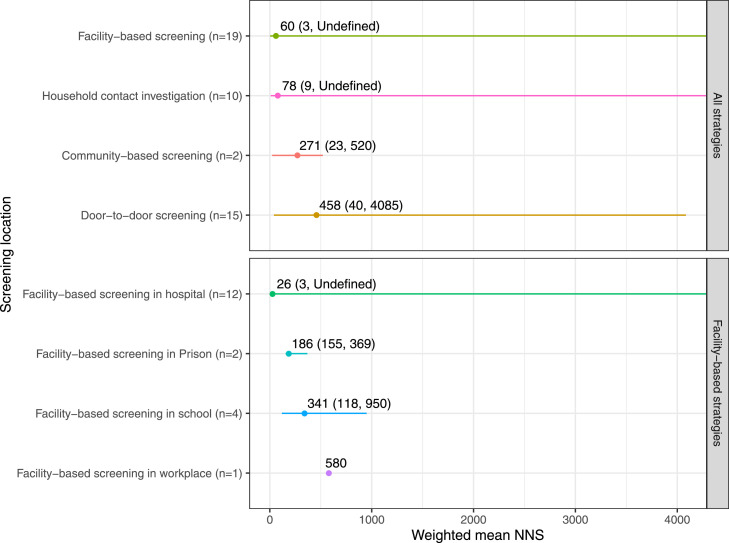
Weighted mean number needed to screen stratified by active case finding screening location. Weighted mean NNS (range) Abbreviations NNS, number needed to screen.

The weighted mean NNS was 271 (23–520, *n*=2) using community-based screening with community health workers or informal health providers,^58^^,^^59^ 458 (40-4085, *n*=15) using door-to-door screening,^18^^,^^19^^,^^21^^,^^25^, ^26^, ^27^^,^^29^^,^^36^^,^^43^^,^ ^48^^,^^49^^,^^53^, ^54^, ^55^^,^^60^ 78 (9-undefined, *n*=10) using household contact investigation,^20^^,^^28^^,^^31^^,^^33^^,^^35^^,^^37^^,^^39^^,^^51^^,^^52^^,^^57^ and 60 (3-undefined, *n*=19) using facility-based screening.^17^^,^^22^, ^23^, ^24^^,^ ^30^^,^^32^^,^^34^^,^^38^^,^^40^, ^41^, ^42^^,^^44^, ^45^, ^46^, ^47^^,^^50^^,^^56^^,^^61^ Amongst facility-based screening methods, weighted mean NNS was 26 (3-undefined, *n*=12) for hospital screening,^17^^,^^22^, ^23^, ^24^^,^^30^^,^^32^^,^^34^^,^ ^38^^,^^40^, ^41^, ^42^^,^^44^, ^45^, ^46^, ^47^^,^^50^^,^^56^^,^^61^ 186 (155–369, *n*=2) in prisons,^30^^,^^50^ 341 (118–950, *n*=4) in schools,^17^^,^^23^^,^^38^ and 580 (*n*=1) in workplaces.^24^ (Figure 4)

### Screening criteria

The weighted mean NNS was 218 (3-undefined, *n*=24) using any symptom as primary screening criteria^20^^,^^22^^,^^23^^,^^24^^,^^26^^,^^27^^,^^35^^,^^39^^,^^41^^,^^42^^,^^44^^,^^46^^,^^48^, ^49^, ^50^, ^51^^,^^53^, ^54^, ^55^^,^^58^, ^59^, ^60^ and 135 (3-undefined, *n*=20) using the WHO symptom screen.^20^^,^^22^^,^^23^^,^^24^^,^^26^^,^^27^^,^^35^^,^^41^^,^^42^^,^^44^^,^^46^^,^^48^, ^49^, ^50^^,^^53^^,^^54^^,^^59^^,^^60^ The weighted mean NNS was 448 (3-undefined, *n*=15) using primary screening criteria of any symptom or an abnormal CXR.^18^^,^^19^^,^^25^^,^^28^, ^29^, ^30^, ^31^, ^32^, ^33^^,^^36^^,^^40^^,^^43^^,^^51^^,^^52^^,^^61^ (Table 2)

**Table 2:** Weighted mean number needed to screen stratified by primary screening criteria.

Primary screening criteria	Weighted mean NNS (Range)	Number of studies
Any symptom	218 (3-undefined)	24
WHO symptom screen	135 (3-undefined)	20
Any symptom or abnormal CXR	448 (3-undefined)	15

Abbreviations

WHO, World Health Organization, CXR, chest X-ray; NNS, number needed to screen

### Loss to follow-up

#### Screening loss to follow-up

After excluding facility-based studies, 12 studies using any symptom as screening criteria, reported data on screening loss to follow-up (proportion of screening eligible people who did not receive screening). Loss-to-follow-up ranged from zero to 33%. The proportion lost to follow-up in screening was 9% (*n*=1) in community-based screening.^59^ The median proportion of loss to follow-up in screening in door-to-door screening was 6% (IQR: 4%, 7%, range: 0–31%, *n*=8)^26^^,^^27^^,^^48^^,^^49^^,^^53^, ^54^, ^55^^,^^60^ and in household contact investigation was 14% (IQR:7%, 23%, range: 0–33%, *n*=3).^20^^,^^35^^,^^39^ (Table 3)

**Table 3:** Proportion of screening loss to follow-up stratified by screening location.

Implementation strategy	Median [IQR]	Range	Number of studies
Community-based screening	9% [-]	(-)	1
Door-to-door screening	6% [4%, 7%]	(0–31%)	8
Household contact investigation	14% [7%, 23%]	(0–33%)	3

Abbreviations

IQR, interquartile range

#### Pre-diagnosis loss to follow-up

Amongst studies which used culture or Xpert for diagnosis in adults, 27 studies reported pre-diagnosis loss to follow-up (the proportion of participants who screened positive but did not receive a confirmatory test). It ranged from zero to 87%. The proportion of pre-diagnosis loss to follow-up was 33% (IQR: 30%, 37%, range: 26–41%, *n*=2) in community-based screening.^58^^,^^59^ The median proportion of pre-diagnosis loss to follow-up in door-to-door screening was 5% (IQR: 3%, 10%, range: 0–65%, n=13)^18^^,^^19^^,^^21^^,^^25^^,^^26^^,^^36^^,^^43^^,^^48^^,^^49^^,^^53^, ^54^, ^55^^,^^60^ and in household contact investigation was 6% (IQR: 4%, 16%, range: 2–26%, *n*=3),^20^^,^^31^^,^^37^ and in facility-based screening was 55% (IQR: 9%, 62%, range: 0–83%, *n*=7). Amongst facility-based screening strategies, median proportion of pre-diagnosis loss to follow-up was 9% (IQR: 0.2%, 67%, range: 0–87%, *n*=6) in hospital^22^^,^^40^^,^^41^^,^^44^^,^^46^^,^^47^; proportion of pre-diagnosis loss to follow-up in prison was 63% (*n*=1),^50^ in school was 55%(*n*=1),^38^ and in workplace was 61% (*n*=1).^24^ (Table 4).

**Table 4:** Proportion of pre-diagnosis loss to follow-up stratified by screening location.

Screening location	Median [IQR]	Range	Number of studies
Community-based screening	33% [30%, 37%]	(26–41%)	2
Door-to-door screening	5% [3%, 10%]	(0–65%)	13
Household contact investigation	6% [4%, 16%]	(2–26%)	3
Facility-based screening	55% [1%, 63%]	(0–87%)	9
Facility-based screening in hospital	9% [0.2%, 67%]	(0–87%)	6
Facility-based screening in Prison	63% [-]	(-)	1
Facility-based screening in school	55% [-]	(-)	1
Facility-based screening in workplace	61% [-]	(-)	1

Abbreviations

IQR, interquartile range.

### Risk of bias

Overall, there was low risk of bias regarding defining target population, choosing appropriate sampling frame and selection of participants, choice of outcome measures, and description of results as per study methods. Several studies either did not report loss to follow-up in the ACF process during screening and diagnosis stage or reported high losses, particularly the pre-diagnosis loss to follow-up. This may have impacted the crude and weighted mean NNS. The study-level results of AXIS risk of bias assessment are presented in supplementary file 4.

## Discussion

We found considerable heterogeneity in reported ACF implementation data across India (2010–2020), including choice of target population, screening location, and screening criteria used. The weighted NNS varied substantially with each of these features. While screening loss to follow-up was uniformly low (less than 33%), pre-diagnosis loss to follow-up was high for some strategies. This review highlights that the yield of ACF in India can vary based on the programmatic choices.

Recommended by the WHO in high-risk populations and settings with high TB prevalence, ACF is a core component of the TB policy of India.^3^^,^^7^^,^^14^ However, a centralized, homogenous ACF implementation strategy is unlikely to deliver on case-finding targets given the expansive geography and diverse population. The reported yield of ACF in the NTEP of India for 2020 was one case identified out of 3290 people screened, or an NNS of 3290.^3^ Such a high NNS makes ACF in NTEP inefficient and costly for a program with already constrained resources. This concern has been repeatedly highlighted by the NTEP in their assessments.^8^^,^^10^ Our results offer insights for how to target ACF programs, indicating the highest yield populations for screening include people with HIV, people with diabetes, tribal populations, and household contacts of people with TB. (Figure 3) The low weighted NNS for certain clinical groups and household contacts are because of their higher risk of TB, resulting in a higher prevalence, thus making ACF more efficient. The rural and tribal populations have more diverse and compounding determinants of TB prevalence, and their low weighted NNS is likely a result of a combination of risk factors and poor access to TB care. While weighted NNS estimates for other groups are lower, the limited number of studies prevents us from drawing any conclusion on such groups.

The results of serial screening in the context of routine TB programs present important insight for the frequency of ACF campaigns. In a rural tribal population in central India with high TB prevalence, the NNS increased from 42 in 2013 to 67 in 2015.^48^ While routine program and background influences on risk factors contributed to the decrease in prevalence, it highlights that repeat ACF campaigns can potentially impact TB burden as increasing NNS over time indicates reduction in prevalence. Although not included in our analysis as it did not meet our review inclusion criteria of year of study, a study from rural Tamil Nadu reported NNS of 183, 248, 353, 278 during prevalence surveys between 1999 and 2001, 2001 and 2003, 2004 and 2006, 2006 and 2008, respectively.^62^ In Vietnam, the Active Case Finding for Tuberculosis 3 (ACT3) trial showed annualized prevalence reduction of 15% per annum through community-wide screening over three years.^63^ In this context, further investigation on optimum frequency of ACF is required.

Facility-based screening and household contact investigation had low weighted NNS estimates in our review. (Figure 4) Amongst all facility-based screening strategies, screening in a hospital setting had the lowest weighted NNS (26, range 3-undefined). Although the community-based screening through community health workers or informal providers had a higher weighted NNS (271, range 23–520), it is difficult to draw any conclusion about the yield of this strategy because it included only two studies. Interestingly, door-to-door screening had a much higher weighted NNS (458, range 40–4085), though this strategy was employed only in populations with known high underlying prevalence of TB. This further emphasizes the point of matching the screening strategy to the appropriate population. Door-to-door screening performed in a low-prevalence setting will have low yield and inefficiencies. Therefore, segmenting populations and a targeted ACF strategy based on feasibility and programmatic considerations is useful. Among the different primary screening criteria used in these studies, the WHO symptom screen had a lower weighted mean NNS compared to screening criteria of any symptom and any symptom combined with abnormal chest x-ray (CXR). Further, Xpert was used for screening only in one study that also used symptom screen and CXR for screening, thus no conclusions can be drawn.^33^ Generally, any symptom or abnormal CXR has a lower NNS than only symptom-based criteria because CXR can detect abnormalities in lungs even before TB symptoms appear. However, the NNS for screening criteria is also influenced by risk group and screening location. Further, the NNS—when calculated from program implementation instead of prevalence surveys—is not just a function of these factors but also affected by loss to follow-up in the ACF cascade.

The proportion of loss to follow-up in screening was low, indicating most people eligible or registered for screening were screened. Overall, about 10% or less were not screened in 12 studies involving community-based, door-to-door, and household contact investigation strategies indicating good coverage. However, in two studies, the screening loss to follow-up in household children contacts and community-wide screening in a rural community was over 30%, but the reason was unclear.^27^^,^^39^ The pre-diagnosis loss to follow-up was more variable and alarmingly high in several studies. The facility-based and community-based screening strategies had a higher pre-diagnosis loss to follow-up than door-to-door screening and household contact investigation strategies. A high screening and pre-diagnosis loss to follow-up in a cascade can artificially inflate NNS because of missing people with active TB. Social, structural, and health system factors responsible for preventing people with presumptive TB from accessing diagnostics.^31^^,^^41^^,^^47^^,^^59^^,^^60^^,^^64^ Loss to follow-up, particularly of people with presumptive TB unable to access diagnostic services, is a missed opportunity. It can be reduced by eliminating social, structural, and health system barriers to improve access to TB care, in addition to ensuring availability of high-quality diagnostics.

The composite NNS of 3290 for the ACF campaign of NTEP in 2020 needs to be contextualized and disaggregated.^3^ The ACF targeted several unique risk groups in different geographies, used WHO symptom screen for screening criteria, and utilized different screening locations, including mobile vans with CXR. However, there has been hardly any operational research that can illuminate valuable information on programmatic NNS in NTEP that disaggregates ACF on various parameters like our review does.^65^ Further, even outside NTEP, the ACF studies are mostly limited to southern and western India. The paucity of data to guide choice of risk groups for screening is an obstacle for NTEP. Coupled with the TB prevalence estimates from the ongoing national prevalence survey, operational estimates of NNS from the NTEP and other ACF implementations will provide data to inform future ACF strategies for India.

A limitation of our study emanates from the heterogeneity in ACF interventions across geographies in India, risk groups, screening location, and screening criteria. Thus, direct comparison of crude NNS estimates was challenging. Instead, we used weighted mean NNS for different risk groups, strategies, and screening criteria and report range of crude NNS. Not all papers in our study reported important outcome indicators necessary to calculate the ACF parameters, and few reported treatment figures. The studies in the future should report the ACF cascade as recommended by the WHO,^66^ thus facilitating estimates of NNS and loss to follow-up in screening, diagnosis, and treatment. We also had to limit the scope of loss to follow-up estimation, as many studies did not report these figures. The pre-diagnosis loss to follow-up analysis was limited to studies using culture or Xpert, both of which are sputum-based tests. Nonetheless, our analysis reflects the reality of programmatic ACF implementation in India and provides valuable estimates for decision making.

In conclusion, we found that ACF implementation is highly variable in India and current literature is sparse for many important risk groups. The NTEP should prioritize ACF in people living with HIV, people with diabetes, and tribal populations, and household contacts of people with TB. However, ACF campaigns should be designed with appropriate consideration to screening location, screening criteria, and other social, structural, and health system factors that can also cause loss to follow-up. Finally, we highlight the need for further research to identify target populations for ACF across India, which is essential to contextualizing ACF and accruing maximum yield.

## Contributors

Conceptualization: TG, LHC, FN, AES, JEG

Data curation: TG, LHC

Analysis: TG

Writing – original draft: TG

Writing – review and edit: TG, LHC, FN, AES, JEG

All authors edited and approved the final manuscript and had access to the raw data. TG and LHC accessed and verified all the data and had final responsibility to submit for publication.

## Data sharing statement

The data used for analysis is available within the paper and supplementary files.

## Editor note

The Lancet Group takes a neutral position with respect to territorial claims in published maps and institutional affiliations.

## Declaration of interests

LHC, FN, and JEG declare funding from the WHO TB Programme for the systematic review of active case finding for TB. TG and JEG are authors of studies included in this systematic review. The views and information presented are our own. The WHO, NIH, and Fulbright-Nehru Master's Fellowship had no role in the conduct of the study or writing the review.

All other authors declare no competing interests.

## Appendix Supplementary materials

Image, application 1

Image, application 2

Image, application 3

Image, application 4

Image, application 5

Image, application 6
