# Supplementary material for: A systematic review and meta-analysis of active case finding for tuberculosis in India
Source: Lancet Reg Health Southeast Asia. 2022 Sep 17;7:100076. doi: 10.1016/j.lansea.2022.100076 (PMC10305973; doi:10.1016/j.lansea.2022.100076)
Supplement: Supplementary file 1 [file mmc1.docx]

Supplementary File 1: Search strategy

Supplementary File 2: PRISMA checklists

Supplementary File 3: Crude estimates of number needed to screen and loss to follow-up

Supplementary File 4: Risk of bias assessment

Supplementary File 5: Additional analysis
