# Supplementary material for: A systematic review and meta-analysis of active case finding for tuberculosis in India
Source: Lancet Reg Health Southeast Asia. 2022 Sep 17;7:100076. doi: 10.1016/j.lansea.2022.100076 (PMC10305973; doi:10.1016/j.lansea.2022.100076)
Supplement: Supplementary file 2 [file mmc2.docx]

## Supplementary File 1: Search strategy

### Previous search

| **Table A.** Systematic review search strategy for previous search |
| --- |
| Search Dates:  Initial search: February 14, 2019  Update: April 13, 2020 |
| PubMed  #1 "tuberculosis"[MeSH Terms]  #2 "tuberculosis"[tw] OR “Pulmonary Consumption”[tw] OR “Consumption, Pulmonary”[tw] OR Phthisis[tw] OR “Tuberculoses”[tw] OR “MDR-TB”[tw] OR “XDR-TB”[tw] OR “MDR TB”[tw] OR “XDR TB”[tw]  #3 #1 OR #2  #4 “Mass Screening”[MeSH Terms] OR “Mass Chest X-Ray”[MeSH Terms] OR "contact tracing"[MeSH Terms] OR “health surveys”[MeSH Terms] OR “Cross-Sectional Studies”[MeSH Terms] OR “Epidemiologic Studies”[MeSH Terms]  #5 “Mass Chest X Ray”[tw] OR “Mass Chest X-Rays”[tw] OR “screenings”[tw] OR “screening”[tw] OR “cross-sectional”[tw] OR “case-detection”[tw] OR “case finding”[tw] OR “contact tracing”[tw] OR “health survey”[tw] OR "prevalence survey"[tw] OR “prevalence studies”[tw] OR "mass radiography"[tw] OR "contact examination"[tw]  #6 #4 OR #5  #7 #3 AND #6  #8 ("animals"[MeSH Terms] NOT ("humans"[MeSH Terms] AND "animals"[MeSH Terms]))  #9 #7 NOT #8  #10 ("2010/11/01"[EDAT] : "3000/12/31"[EDAT] OR "2010/11/01"[CRDT] : "3000/12/31"[CRDT]) OR ("2010/11/01"[PDAT] : "3000/11/31"[PDAT])  #11 #9 AND #10 |
| Embase  #1 'tuberculosis'/exp OR 'lung tuberculosis'/exp  #2 (‘tuberculosis’ OR ‘Pulmonary Consumption’ OR ‘Consumption, Pulmonary’ OR Phthisis OR ‘Tuberculoses’ OR “MDR-TB” OR “XDR-TB” OR “MDR TB” OR “XDR TB”):ab,ti,kw  #3 #1 OR #2  #4 'tuberculosis control'/exp OR 'case finding'/exp OR 'mass radiography'/exp OR 'mass screening'/exp OR 'contact examination'/exp OR 'screening'/exp  #5 (‘Mass Chest X Ray’ OR ‘Mass Chest X-Rays’ OR ‘Screenings’ OR ‘screening’ OR ‘Cross-Sectional Studies’ OR ‘Case-detection’ OR ‘case finding’ OR ‘contact tracing’ OR ‘mass radiography’ OR ‘contact examination’ OR ‘health survey’ OR ‘cross-sectional’ OR 'prevalence survey' OR ‘prevalence studies’):ab,ti,kw  #6 #4 OR #5  #7 #3 AND #6  #8 'animal'/exp NOT ('animal'/exp AND 'human'/exp)  #9 #7 NOT #8  #10 [1-11-2010]/sd  #11 #9 AND #10 |
| Scopus  #1 TITLE-ABS-KEY (tuberculosis OR phthisis OR "pulmonary consumption" OR Tuberculoses OR “MDR-TB” OR “XDR-TB” OR “MDR TB” OR “XDR TB”)  #2 TITLE-ABS-KEY("mass chest x ray" OR "mass chest x-rays" OR screenings OR screening OR "health survey" OR "cross-sectional" OR "case-detection" OR "case finding" OR "contact tracing" OR "prevalence survey" OR "prevalence studies" OR "mass radiography" OR "contact examination")  #3 #1 AND #2  #4 PUBDATETXT ( november 2010 ) OR PUBDATETXT ( december 2010 ) OR PUBYEAR > 2010  #5 #3 and #4 |
| Cochrane Library  #1 MeSH descriptor: [Tuberculosis] explode all trees  #2 "tuberculosis" OR (Pulmonary NEXT Consumption*) OR Phthisis OR Tuberculoses OR “MDR-TB” OR “XDR-TB” OR “MDR TB” OR “XDR TB”  #3 #1 OR #2  #4 MeSH descriptor: [Mass Screening] explode all trees  #5 MeSH descriptor: [Mass Chest X-Ray] explode all trees  #6 MeSH descriptor: [Contact Tracing] explode all trees  #7 MeSH descriptor: [Health Surveys] explode all trees  #8 MeSH descriptor: [Cross-Sectional Studies] explode all trees  #9 MeSH descriptor: [Epidemiologic Studies] explode all trees  #10 “Mass Chest X Ray” OR “Mass Chest X-Rays” OR “screenings” OR “screening” OR “cross-sectional” OR “case-detection” OR “case finding” OR “contact tracing” OR “health survey” OR "prevalence survey" OR “prevalence studies” OR "mass radiography" OR "contact examination"  #11 {OR #4-#10}  #12 #3 AND #11 with Cochrane Library publication date Between Nov 2010 and Mar 2019 |

### New search

| **Table B.** Systematic review search strategy for new search |
| --- |
| Search Dates:  Initial search: December 18, 2020 |
| PubMed  #1 "tuberculosis"[MeSH Terms]  #2 "tuberculosis"[tw] OR “Pulmonary Consumption”[tw] OR “Consumption, Pulmonary”[tw] OR Phthisis[tw] OR “Tuberculoses”[tw] OR “MDR-TB”[tw] OR “XDR-TB”[tw] OR “MDR TB”[tw] OR “XDR TB”[tw]  #3 #1 OR #2  #4 “Mass Screening”[MeSH Terms] OR “Mass Chest X-Ray”[MeSH Terms] OR "contact tracing"[MeSH Terms] OR “health surveys”[MeSH Terms] OR “Cross-Sectional Studies”[MeSH Terms] OR “Epidemiologic Studies”[MeSH Terms]  #5 “Mass Chest X Ray”[tw] OR “Mass Chest X-Rays”[tw] OR “screenings”[tw] OR “screening”[tw] OR “cross-sectional”[tw] OR “case-detection”[tw] OR “case finding”[tw] OR “contact tracing”[tw] OR “health survey”[tw] OR "prevalence survey"[tw] OR “prevalence studies”[tw] OR "mass radiography"[tw] OR "contact examination"[tw]  #6 #4 OR #5  #7 #3 AND #6  #8 ("animals"[MeSH Terms] NOT ("humans"[MeSH Terms] AND "animals"[MeSH Terms]))  #9 #7 NOT #8  #10 ("2020/01/01"[EDAT] : "3000/12/31"[EDAT] OR "2020/01/01"[CRDT] : "3000/12/31"[CRDT]) OR ("2020/01/01"[PDAT] : "3000/11/31"[PDAT])  #11 #9 AND #10  #12 "India"[Mesh] OR Rajasthan[tw] OR "Madhya Pradesh"[tw] OR Maharashtra[tw] OR "Uttar Pradesh"[tw] OR Gujarat[tw] OR Karnataka[tw] OR Ladakh[tw] OR "Andhra Pradesh"[tw] OR Odisha[tw] OR Chhattisgarh[tw] OR "Tamil Nadu"[tw] OR Telangana[tw] OR Bihar[tw] OR "West Bengal"[tw] OR "Arunachal Pradesh"[tw] OR Jharkhand[tw] OR Assam[tw] OR "Himachal Pradesh"[tw] OR "Jammu and Kashmir"[tw] OR Uttarakhand[tw] OR Punjab[tw] OR Haryana[tw] OR Kerala[tw] OR Meghalaya[tw] OR Manipur[tw] OR Mizoram[tw] OR Nagaland[tw] OR Tripura[tw] OR "Andaman and Nicobar Islands"[tw] OR Sikkim[tw] OR Goa[tw] OR Delhi[tw] OR "Dadra and Nagar Haveli"[tw] OR "Daman and Diu"[tw] OR Puducherry[tw] OR Chandigarh[tw] OR Lakshadweep[tw]  #13 #11 AND #12 |
| Embase  #1 'tuberculosis'/exp OR 'lung tuberculosis'/exp  #2 (‘tuberculosis’ OR ‘Pulmonary Consumption’ OR ‘Consumption, Pulmonary’ OR Phthisis OR ‘Tuberculoses’ OR “MDR-TB” OR “XDR-TB” OR “MDR TB” OR “XDR TB”):ab,ti,kw  #3 #1 OR #2  #4 'tuberculosis control'/exp OR 'case finding'/exp OR 'mass radiography'/exp OR 'mass screening'/exp OR 'contact examination'/exp OR 'screening'/exp  #5 (‘Mass Chest X Ray’ OR ‘Mass Chest X-Rays’ OR ‘Screenings’ OR ‘screening’ OR ‘Cross-Sectional Studies’ OR ‘Case-detection’ OR ‘case finding’ OR ‘contact tracing’ OR ‘mass radiography’ OR ‘contact examination’ OR ‘health survey’ OR ‘cross-sectional’ OR 'prevalence survey' OR ‘prevalence studies’):ab,ti,kw  #6 #4 OR #5  #7 #3 AND #6  #8 'animal'/exp NOT ('animal'/exp AND 'human'/exp)  #9 #7 NOT #8  #10 [18-12-2020]/sd  #11 #9 AND #10  #12 'India'/exp OR (Rajasthan OR "Madhya Pradesh" OR Maharashtra OR "Uttar Pradesh" OR Gujarat OR Karnataka OR Ladakh OR "Andhra Pradesh" OR Odisha OR Chhattisgarh OR "Tamil Nadu" OR Telangana OR Bihar OR "West Bengal" OR "Arunachal Pradesh" OR Jharkhand OR Assam OR "Himachal Pradesh" OR "Jammu and Kashmir" OR Uttarakhand OR Punjab OR Haryana OR Kerala OR Meghalaya OR Manipur OR Mizoram OR Nagaland OR Tripura OR "Andaman and Nicobar Islands" OR Sikkim OR Goa OR Delhi OR "Dadra and Nagar Haveli" OR "Daman and Diu" OR Puducherry OR Chandigarh OR Lakshadweep):ab,ti,kw  #13 #12 AND #13 |
| Scopus  #1 TITLE-ABS-KEY (tuberculosis OR phthisis OR "pulmonary consumption" OR Tuberculoses OR “MDR-TB” OR “XDR-TB” OR “MDR TB” OR “XDR TB”)  #2 TITLE-ABS-KEY("mass chest x ray" OR "mass chest x-rays" OR screenings OR screening OR "health survey" OR "cross-sectional" OR "case-detection" OR "case finding" OR "contact tracing" OR "prevalence survey" OR "prevalence studies" OR "mass radiography" OR "contact examination")  #3 #1 AND #2  #4 PUBYEAR > 2019  #5 #3 and #4  #6 TITLE-ABS-KEY (India OR Rajasthan OR "Madhya Pradesh" OR Maharashtra OR "Uttar Pradesh" OR Gujarat OR Karnataka OR Ladakh OR "Andhra Pradesh" OR Odisha OR Chhattisgarh OR "Tamil Nadu" OR Telangana OR Bihar OR "West Bengal" OR "Arunachal Pradesh" OR Jharkhand OR Assam OR "Himachal Pradesh" OR "Jammu and Kashmir" OR Uttarakhand OR Punjab OR Haryana OR Kerala OR Meghalaya OR Manipur OR Mizoram OR Nagaland OR Tripura OR "Andaman and Nicobar Islands" OR Sikkim OR Goa OR Delhi OR "Dadra and Nagar Haveli" OR "Daman and Diu" OR Puducherry OR Chandigarh OR Lakshadweep)  #7 #5 AND #6 |
| Cochrane Library  #1 MeSH descriptor: [Tuberculosis] explode all trees  #2 "tuberculosis" OR (Pulmonary NEXT Consumption*) OR Phthisis OR Tuberculoses OR “MDR-TB” OR “XDR-TB” OR “MDR TB” OR “XDR TB”  #3 #1 OR #2  #4 MeSH descriptor: [Mass Screening] explode all trees  #5 MeSH descriptor: [Mass Chest X-Ray] explode all trees  #6 MeSH descriptor: [Contact Tracing] explode all trees  #7 MeSH descriptor: [Health Surveys] explode all trees  #8 MeSH descriptor: [Cross-Sectional Studies] explode all trees  #9 MeSH descriptor: [Epidemiologic Studies] explode all trees  #10 “Mass Chest X Ray” OR “Mass Chest X-Rays” OR “screenings” OR “screening” OR “cross-sectional” OR “case-detection” OR “case finding” OR “contact tracing” OR “health survey” OR "prevalence survey" OR “prevalence studies” OR "mass radiography" OR "contact examination"  #11 {OR #4-#10}  #12 #3 AND #11 with Cochrane Library publication date Between Jan 2020 and Dec 2020 |
