# Supplementary material for: A systematic review and meta-analysis of active case finding for tuberculosis in India
Source: Lancet Reg Health Southeast Asia. 2022 Sep 17;7:100076. doi: 10.1016/j.lansea.2022.100076 (PMC10305973; doi:10.1016/j.lansea.2022.100076)
Supplement: Supplementary file 4 [file mmc4.docx]

# Supplementary File 3: Crude estimates of number needed to screen and loss to follow-up

| A | B | C | D | E | F | G | H | I | J | K | L | M (= K - L) | N (= M / K) | O | P | Q (= O - P) | R (= Q / O) | S | T (= L / S) |
| --- | --- | --- | --- | --- | --- | --- | --- | --- | --- | --- | --- | --- | --- | --- | --- | --- | --- | --- | --- |
| Author, Year | State | Population | Health sector | Health system level | Risk group | Screening location | Primary screening criteria | Type of TB | Diagnostic criteria | Number eligible for screening | Number screened | Screening loss to follow-up | | Number eligible for testing | Number tested | Pre-diagnosis loss to follow-up | | Number of TB cases | Number needed to screen |
|  |  |  |  |  |  |  |  |  |  |  |  | Number | Proportion |  |  | Number | Proportion |  |  |
| Dahiwale, 2011 | MP | Mixed | Public | Tertiary | Household contact, Household child contact | Facility-based screening in hospital | TST, CXR, Clinical examination | Both | TST | NA | 168 | NA | NA | NA | NA | NA | NA | 23 | 7 |
| Gupta, 2011 | MH | Mixed | Public | Tertiary | People with HIV | Facility-based screening in hospital | WHO symptom screen, TST | PTB | Culture | 840 | 799 | 41 | 5% | 130 | 107 | 23* | 18% | 9 | 89 |
| Pothukuchi, 2011 | AP | Unclear | Public | Primary | Household contact, Household child contact | Household contact investigation | Symptom | PTB | Smear | 172 | 116 | 56^ | 33% | NA | NA | NA | NA | 0 | Undefined |
| Chadha, 2012 | KA | Rural | Public | Primary | Community-wide | Door-to-door screening | WHO symptom screen, CXR | PTB | Culture | 71874 | 63362 | 8512 | 12% | 5120 | 4850 | 270* | 5% | 86 | 737 |
| Bhat, 2013 | KA | Mixed | Public | Mixed | Malnourished children | Facility-based screening in hospital | TST, CXR, Smear | PTB | Smear, TST, CXR | 1927 | 1927 | 0 | 0% | 1927 | 1173 | 754 | 39% | 19 | 101 |
| Chauhan, 2013 | UP | Mixed | Private | Tertiary | Household contact, Household child contact | Facility-based screening in hospital | WHO symptom screen, CXR, TST | Both | CXR | 253 | 200 | 53 | 21% | NA | NA | NA | NA | 95 | 2 |
| Rekha Devi, 2013 | Multiple: ARP, AS | Rural | Public | Primary | Community-wide | Door-to-door screening | Cough ≥ 1 week | PTB | Smear | NA | 2187 | NA | NA | 704 | 248 | 456* | 65% | 4 | 547 |
| Rekha, 2013 | TN | Mixed | Public | Primary | Household contact, Household child contact | Household contact investigation | WHO symptom screen, CXR, TST | PTB | Clinical, CXR, TST | 87 | 53 | 34 | 39% | 53 | 53 | 0 | 0% | 0 | Undefined |
| Singh, 2013 | DL | Mixed | Public | Tertiary | Household contact | Household contact investigation | Smear, Culture | PTB | Culture | 1792 | 1608 | 184 | 10% | NA | 1206 | NA | NA | 52 | 31 |
| Chatterjee, 2014 | TN | Rural | Public | Primary | Community-wide | Door-to-door screening | WHO symptom screen | PTB | Culture | 7397 | 5096 | 2301^ | 31% | NA | NA | NA | NA | 25 | 204 |
| Isaakidis, 2014 | MH | Urban | Public-Private mix | Mixed | People with HIV | Facility-based screening in hospital | WHO symptom screen | Both | Culture | NA | 14135 | NA | NA | 1741 | 1724 | 17* | 1% | 202 | 70 |
| Aggarwal, 2015 | PB | Mixed | Public | Primary | Community-wide | Door-to-door screening | WHO symptom screen | PTB | Culture | 91030 | 85770 | 5260^ | 6% | 2898 | 2821 | 77* | 3% | 21 | 4084 |
| Dhanaraj, 2015 | TN | Urban | Public | Primary | Community-wide | Door-to-door screening | WHO symptom screen, CXR | PTB | Culture | 59957 | 55617 | 4340 | 7% | 6139 | 5373 | 766* | 12% | 126 | 441 |
| Jada, 2015 | TN | Rural | Public | Primary | Community-wide | Door-to-door screening | WHO symptom screen | PTB | Culture | 1510 | 1510 | 0^ | 0% | 126 | 126 | 0* | 0% | 31 | 49 |
| Jain, 2015 | MH | Mixed | Public | Tertiary | People with HIV | Facility-based screening in hospital | CXR, Smear, Culture | PTB | Culture | NA | 263 | NA | NA | 263 | NA | NA | NA | 28 | 9 |
| Narang, 2015 | MH | Mixed | Public | Tertiary | Community-wide | Door-to-door screening | WHO symptom screen, CXR | PTB | Culture | 55096 | 50332 | 4764 | 9% | 4805 | 4312 | 493* | 10% | 66 | 763 |
| Rao, 2015 | MP | Rural | Public | Primary | Tribal population | Door-to-door screening | WHO symptom screen | PTB | Culture | 10259 | 9653 | 606^ | 6% | 1100 | 1071 | 29* | 3% | 243 | 40 |
| Sharma, 2015 | HR | Mixed | Public | Primary | Community-wide | Door-to-door screening | WHO symptom screen | PTB | Culture | 105202 | 98599 | 6603^ | 6% | 1860 | 1337 | 523* | 28% | 82 | 1202 |
| Dierberg, 2016 | Multiple: KA, HP, UK | Mixed | Public | Mixed | Tibetan population | Facility-based screening in school | WHO symptom screen | Both | Xpert | NA | 27714 | NA | NA | NA | 3830 | NA | NA | 65 | 426 |
| Nair, 2016 | TN | Unclear | Public | Unclear | Household contact | Household contact investigation | CXR, Symptom | PTB | Culture | 643 | 544 | 99 | 15% | 50 | NA | NA | NA | 26 | 21 |
| Padmapriyadarsini, 2016 | Multiple | Mixed | Public | Unclear | People with HIV | Facility-based screening in hospital | WHO symptom screen | Both | Smear | NA | 1662 | NA | NA | 434 | 57 | 377* | 87% | 23 | 72 |
| Uppada, 2016 | AP | Mixed | Public | Primary | School-going adolescent | Facility-based screening in school | WHO symptom screen, TST | PTB | Culture | 12388 | 6643 | 5745 | 46% | 1314 | 589 | 725* | 55% | 7 | 949 |
| Dolla, 2017 | TN | Urban | Public | Primary | Homeless population | Door-to-door screening | WHO symptom screen, CXR | PTB | Culture | 332 | 301 | 31 | 9% | NA | NA | NA | NA | 3 | 100 |
| Mave, 2017 | MH | Mixed | Public | Tertiary | People with Diabetes | Facility-based screening in hospital | WHO symptom screen | PTB | Culture | 630 | 630 | 0 | 0% | 111 | 111 | 0* | 0% | 0 | Undefined |
| Mazahir, 2017 | UP | Mixed | Public | Tertiary | Household contact, Household child contact | Household contact investigation | WHO symptom screen, CXR, TST, AFB | Both | Clinical, CXR, Histopathology | 80 | 80 | 0 | 0% | NA | NA | NA | NA | 9 | 9 |
| Chatla, 2018 | Multiple: AP, TN | Mixed | Public | Secondary | Household contact | Household contact investigation | WHO symptom screen | PTB | Xpert | 4771 | 4771 | 0^ | 0% | 793 | 781 | 12* | 2% | 34 | 140 |
| Dutta, 2018 | OR | Urban | Public | Primary | Migrant population, Slum residents | Community-based screening | Cough ≥ 2 weeks | PTB | Xpert | NA | 253679 | NA | NA | 3780 | 2800 | 980* | 26% | 488 | 520 |
| Ranganath, 2018 | KA | Mixed | Public | Primary | Household contact, Household child contact | Household contact investigation | WHO symptom screen | PTB | Not defined | 110 | 95 | 15^ | 14% | NA | NA | NA | NA | 2 | 48 |
| Sarin, 2018 | DL | Urban | Public | Primary | Slum residents | Door-to-door screening | Cough ≥2 weeks *OR* Any cough and History of contact | PTB | Culture | 40756 | 40529 | 227^ | 1% | 691 | 654 | 37* | 5% | 49 | 827 |
| Bhatnagar, 2019 | MZ | Mixed | Public | Secondary | Incarcerated persons | Facility-based screening in Prison | WHO symptom screen | PTB | Xpert | 2397 | 738 | 1659 | 69% | 391 | 145 | 246* | 63% | 2 | 369 |
| Chadha, 2019 | Multiple: TN, MH, KA, GJ, HR, MP, UP, PB | Mixed | Public | Mixed | Community-wide | Door-to-door screening | WHO symptom screen, CXR | PTB | Culture | 769290 | 715989 | 53301 | 7% | 55608 | 50852 | 4756* | 9% | 1346 | 532 |
| Chadha, 2019 | Multiple: GJ, MH, TN, KA | Mixed | Public | Mixed | Community-wide | Door-to-door screening | WHO symptom screen, CXR | PTB | Culture | 333614 | 305913 | 27701 | 8% | 33144 | 29990 | 3154* | 10% | 839 | 365 |
| Dolla, 2019 | TN | Urban | Public | Primary | Incarcerated people | Facility-based screening in Prison | WHO symptom screen, CXR | PTB | Culture | NA | 1854 | NA | NA | NA | NA | NA | NA | 12 | 155 |
| Dorjee, 2019 | HP | Unclear | Public | Primary | Tibetan population, School-going adolescent | Facility-based screening in school | WHO symptom screen | Both | Xpert | NA | 5391 | NA | NA | NA | NA | NA | NA | 46 | 117 |
|  | HP | Unclear | Public | Primary | Tibetan population | Facility-based screening in school | WHO symptom screen | Both | Xpert | NA | 786 | NA | NA | NA | NA | NA | NA | 1 | 786 |
| Dravid, 2019 | MH | Urban | Private | Tertiary | People with HIV | Facility-based screening in hospital | WHO symptom screen | Both | Culture | 1904 | 1904 | 0 | 0% | NA | NA | NA | NA | 637 | 3 |
| Rao, 2019 | MP | Rural | Public | Primary | Tribal population | Door-to-door screening | WHO symptom screen | PTB | Culture | 10300 | 9756 | 544^ | 5% | 1463 | 1430 | 33* | 2% | 232 | 42 |
| Shriraam, 2019 | TN | Rural | Public | Primary | Migrant population, Brick kiln workers | Facility-based screening in workplace | WHO symptom screen | PTB | Xpert | 650 | 580 | 70 | 11% | 56 | 22 | 34* | 61% | 1 | 580 |
| Sireesha, 2019 | TL | Unclear | Public | Tertiary | People with Diabetes | Facility-based screening in hospital | CXR, Symptom | PTB | Culture | 500 | 500 | 0 | 0% | 200 | 200 | 0* | 0% | 24 | 21 |
| Vijayageetha, 2019 | PY | Urban | Public | Tertiary | Pregnant women | Facility-based screening in hospital | WHO symptom screen | Both | Xpert, Culture | 4203 | 4203 | 0 | 0% | 77 | 13 | 64* | 83% | 1 | 4203 |
| Ananthakrishnan, 2020 | TN | Urban | Public-Private mix | Primary | Household contact | Household contact investigation | WHO symptom screen, CXR | PTB | Xpert | 7458 | 5553 | 1905 | 26% | 1312 | 971 | 341* | 26% | 35 | 159 |
| Bekken, 2020 | AP | Unclear | Public | Unclear | Household contact | Household contact investigation | Culture | PTB | Culture | 557 | 525 | 32 | 6% | 525 | 493 | 32* | 6% | 29 | 18 |
| Dabhi, 2020 | TN | Mixed | Private | Tertiary | People with Diabetes | Facility-based screening in hospital | WHO symptom screen, CXR | Both | Culture | NA | 1000 | NA | NA | 50 | 50 | NA | 0% | 9 | 111 |
| Garg, 2020 | BR | Rural | Public | Primary | Community-wide | Community-based screening | WHO symptom screen | Both | Xpert | 12394 | 11223 | 1171^ | 9% | 9895 | 5864 | 4031* | 41% | 492 | 23 |
| Hussain, 2020 | OR | Rural | Public | Primary | Tribal population | Door-to-door screening | WHO symptom screen | PTB | Culture | 5750 | 5144 | 606^ | 11% | 126 | 126 | 0* | 0% | 18 | 286 |
| Velayutham, 2020 | TN | Urban | Public | Primary | Household contact | Household contact investigation | WHO symptom screen, CXR, AFB, Xpert | Both | Xpert | 2687 | 2150 | 537 | 20% | NA | NA | NA | NA | 13 | 165 |

*Abbreviations*

***States***: AP: Andhra Pradesh, ARP: Arunachal Pradesh, AS: Assam, BR: Bihar, DL: Delhi, GJ: Gujarat, HR: Haryana, HP: Himachal Pradesh, KA: Karnataka, MP: Madhya Pradesh, MH: Maharashtra, MZ: Mizoram, OR: Orissa, PY: Pondicherry, PB: Punjab, TN: Tamil Nadu, TL: Telangana, UP: Uttar Pradesh, UK: Uttarakhand; ***Primary screening criteria***: CXR: chest X-ray, TST: Tuberculin Skin Testing, AFB: Sputum smear; ***Type of TB***: PTB: pulmonary TB, EPTB: Extra-pulmonary TB; NA: Not available. **^**Studies included in the screening loss to follow-up analysis: primary screening criteria of any symptom excluding any studies using facility-based screening strategies (n = 12). ***** Studies included in the pre-diagnosis loss to follow-up analysis: diagnosis using culture or Xpert in adult population (n = 27).
