# Supplementary material for: A systematic review and meta-analysis of active case finding for tuberculosis in India
Source: Lancet Reg Health Southeast Asia. 2022 Sep 17;7:100076. doi: 10.1016/j.lansea.2022.100076 (PMC10305973; doi:10.1016/j.lansea.2022.100076)
Supplement: Supplementary file 5 [file mmc5.docx]

# Supplementary File 4: Risk of bias assessment

| Serial | Author, Year | 4  Was the target/reference population clearly defined? | 5  Was the sample frame taken from an appropriate population base so that it closely represented the target/reference population under investigation? | 6  Was the selection process likely to select subjects/participants that were representative of the target/reference population under investigation? | 7  Were measures undertaken to address and categorise non-responders? | 8  Were the risk factor and outcome variables measured appropriate to the aims of the study? | 9  Were the risk factor and outcome variables measured correctly using instruments/measurements that had been trialled, piloted or published previously? | 12  Were the basic data adequately described? | 13  Does the response rate raise concerns about non-response bias? | 14  If appropriate, was information about non-responders described? | 15  Were the results internally consistent? | 16  Were the results for the analyses described in the methods, presented? | 19  Were there any funding sources or conflicts of interest that may affect the authors’ interpretation of the results? | 20  Was ethical approval or consent of participants attained? |
| --- | --- | --- | --- | --- | --- | --- | --- | --- | --- | --- | --- | --- | --- | --- |
| 1 | Chadha, 2019 | Yes | Yes | Yes | Yes | Yes | Yes | Yes | No | NA | Yes | Yes | No | Yes |
| 2 | Rao, 2019 | Yes | Yes | Yes | Yes | Yes | Yes | Yes | No | NA | Yes | Yes | No | Yes |
| 3 | Hussain, 2020 | Yes | Yes | Yes | Yes | Yes | Yes | Yes | No | NA | Yes | Yes | No | Yes |
| 4 | Chadha, 2019 | Yes | Yes | Yes | No | Yes | Yes | Yes | No | NA | Yes | Yes | No | Yes |
| 5 | Bekken, 2020 | Yes | Yes | Yes | No | Yes | Yes | Yes | No | NA | Yes | Yes | No | Yes |
| 6 | Chatla, 2018 | Yes | Yes | Yes | Yes | Yes | Yes | Yes | No | NA | Yes | Yes | No | Yes |
| 7 | Dravid, 2019 | Yes | Yes | Yes | Don't know | Yes | Yes | Yes | Don't know | NA | Yes | Yes | No | Yes |
| 8 | Shriraam, 2019 | Yes | Yes | Yes | No | Yes | Yes | Yes | Yes | No | Yes | Yes | No | Yes |
| 9 | Vijayageetha, 2019 | Yes | Yes | Yes | Yes | Yes | Yes | Yes | Yes | No | Yes | Yes | No | Yes |
| 10 | Bhatnagar, 2019 | Yes | Yes | Yes | No | Yes | Yes | Yes | Yes | No | Yes | Yes | No | Yes |
| 11 | Bhat, 2013 | Yes | Yes | Yes | No | Yes | Yes | Yes | Yes | No | Yes | Yes | No | Yes |
| 12 | Uppada, 2016 | Yes | Yes | Yes | Yes | Yes | Yes | Yes | Yes | Yes | Yes | Yes | No | Yes |
| 13 | Rekha, 2013 | Yes | Yes | Yes | Yes | Yes | Yes | Yes | Yes | Yes | Yes | Yes | No | Yes |
| 14 | Chauhan, 2013 | Yes | Yes | Yes | No | Yes | Yes | Yes | Yes | No | Yes | Yes | No | Yes |
| 15 | Ranganath, 2018 | Yes | Yes | Yes | Yes | Yes | Yes | Yes | Don't know | NA | Yes | Yes | No | Yes |
| 16 | Mazahir, 2017 | Yes | Yes | Don't know | Yes | Yes | Yes | Yes | Don't know | NA | Yes | Yes | No | Yes |
| 17 | Dorjee, 2019 | Yes | Yes | Yes | Don't know | Yes | Yes | Yes | Don't know | No | Yes | Yes | No | Yes |
| 18 | Rao, 2015 | Yes | Yes | Yes | Yes | Yes | Yes | Yes | No | NA | Yes | Yes | No | Yes |
| 19 | Dhanaraj, 2015 | Yes | Yes | Yes | Yes | Yes | Yes | Yes | No | NA | Yes | Yes | No | Yes |
| 20 | Aggarwal, 2015 | Yes | Yes | Yes | Yes | Yes | Yes | Yes | No | NA | Yes | Yes | No | Yes |
| 21 | Jada, 2015 | Yes | Yes | Yes | Yes | Yes | Yes | Yes | No | NA | Yes | Yes | No | Yes |
| 22 | Sarin, 2018 | Yes | Yes | Yes | Yes | Yes | Yes | Yes | No | NA | Yes | Yes | No | Yes |
| 23 | Sharma, 2015 | Yes | Yes | Yes | Yes | Yes | Yes | Yes | No | NA | Yes | Yes | No | Yes |
| 24 | Narang, 2019 | Yes | Yes | Yes | Yes | Yes | Yes | Yes | No | NA | Yes | Yes | No | Yes |
| 25 | Chadha, 2012 | Yes | Yes | Yes | Yes | Yes | Yes | Yes | No | NA | Yes | Yes | No | Yes |
| 26 | Chatterjee, 2014 | Yes | Yes | Yes | Yes | Yes | Yes | Yes | No | NA | Yes | Yes | No | Yes |
| 27 | Rekha Devi, 2013 | Yes | Yes | Yes | Don't know | Yes | Yes | Yes | Yes | No | Yes | Yes | No | Yes |
| 28 | Nair, 2016 | Yes | Yes | Yes | Don't know | Yes | Yes | Yes | Don't know | NA | Yes | Yes | No | Yes |
| 29 | Dahiwale, 2011 | Yes | Yes | Yes | Don't know | Yes | Yes | Yes | Don't know | NA | Yes | Yes | No | Yes |
| 30 | Singh, 2013 | Yes | Yes | Yes | Yes | Yes | Yes | Yes | No | NA | Yes | Yes | No | Yes |
| 31 | Pothukuchi, 2011 | Yes | Yes | Yes | Yes | Yes | Yes | Yes | No | NA | Yes | Yes | No | Yes |
| 32 | Mave, 2017 | Yes | Yes | Yes | Yes | Yes | Yes | Yes | No | NA | Yes | Yes | No | Yes |
| 33 | Sireesha, 2019 | Yes | Yes | Yes | Yes | Yes | Yes | Yes | No | NA | Yes | Yes | No | Yes |
| 34 | Dutta, 2018 | Yes | Yes | Yes | Yes | Yes | Yes | Yes | No | NA | Yes | Yes | No | Yes |
| 35 | Jain, 2015 | Yes | Yes | Yes | No | Yes | Yes | Yes | Don't know | NA | Yes | Yes | No | Yes |
| 36 | Isaakidis, 2014 | Yes | Yes | Yes | Yes | Yes | Yes | Yes | No | NA | Yes | Yes | No | Yes |
| 37 | Padmapriyadarsini, 2016 | Yes | Yes | Yes | No | Yes | Yes | Yes | Yes | No | Yes | Yes | No | Yes |
| 38 | Gupta, 2011 | Yes | Yes | Yes | Yes | Yes | Yes | Yes | No | NA | Yes | Yes | No | Yes |
| 39 | Dolla, 2017 | Yes | Yes | Yes | Yes | Yes | Yes | Yes | No | NA | Yes | Yes | No | Yes |
| 40 | Dolla, 2018 | Yes | Yes | Yes | Don't know | Yes | Yes | Yes | Don't know | NA | Yes | Yes | No | Yes |
| 41 | Dierberg, 2016 | Yes | Yes | Yes | Don't know | Yes | Yes | Yes | Don't know | NA | Yes | Yes | No | Yes |
| 42 | Ananthakrishnan, 2020 | Yes | Yes | Yes | Yes | Yes | Yes | Yes | Yes | No | Yes | Yes | No | Yes |
| 43 | Garg, 2020 | Yes | Yes | Yes | Yes | Yes | Yes | Yes | Yes | No | Yes | Yes | No | Yes |
| 44 | Dabhi, 2020 | Yes | Yes | Yes | Don't know | Yes | Yes | Yes | No | NA | Yes | Yes | No | Yes |
| 45 | Velayutham, 2020 | Yes | Yes | Yes | Yes | Yes | Yes | Yes | No | NA | Yes | Yes | No | Yes |

*Abbreviations*: NA, not applicable

*Legend*: Green indicates low risk of bias, yellow indicates unknown risk of bias, red indicates higher risk of bias. Six AXIS questions that were not relevant for the NNS review were not included: 1) Were the aims/objectives of the study clear? 2) Was the study design appropriate for the stated aims? 3) Was the sample size justified? 10) Is it clear what was used to determine statistical significance and/or precision estimates? 11)  Were the methods (including statistical methods) sufficiently described to enable them to be repeated? 17) Were the authors’ discussions and conclusions justified by the results? 18) Were the limitations of the study discussed?
