# Supplementary material for: A systematic review and meta-analysis of active case finding for tuberculosis in India
Source: Lancet Reg Health Southeast Asia. 2022 Sep 17;7:100076. doi: 10.1016/j.lansea.2022.100076 (PMC10305973; doi:10.1016/j.lansea.2022.100076)
Supplement: Supplementary file 6 [file mmc6.docx]

# Supplementary File 5: Additional analysis

## Table A: Weighted mean number needed to screen stratified by risk group

| Category | Risk group | Weighted mean NNS (Range) | Number of studies |
| --- | --- | --- | --- |
| Clinical | People with HIV | 21 (3-89) | 5 |
|  | People with Diabetes | 65 (21-undefined) | 3 |
|  | Malnourished children | 102 | 1 |
|  | Pregnant women | 4203 | 1 |
| TB contacts | Household child contact | 6 (3-undefined) | 6 |
|  | Household contact | 50 (3-undefined) | 12 |
| Social | Tribal population | 50 (40-286) | 3 |
|  | Homeless population | 101 | 1 |
|  | Incarcerated persons | 186 (155-369) | 2 |
|  | School-going adolescent | 228 (118-950) | 2 |
|  | Tibetan population | 303 (118-786) | 3 |
|  | Migrant population | 520 (520-580) | 2 |
|  | Slum residents | 548 (520-828) | 2 |
|  | Brick kiln workers | 580 | 1 |
| Community-wide | Rural | 131 (23-737) | 5 |
|  | Urban | 442 | 1 |
|  | Mixed | 534 (365-4085) | 5 |

Abbreviations

NNS, number needed to screen

## Table B: Weighted mean number needed to screen stratified by screening location

| Screening location | Weighted mean NNS (Range) | Number of studies |
| --- | --- | --- |
| Community-based screening | 271 (23-520) | 2 |
| Door-to-door screening | 458 (40-4085) | 15 |
| Household contact investigation | 78 (9-undefined) | 10 |
| Facility-based screening | 60 (3-undefined) | 19 |
| Facility-based screening in hospital | 26 (3-undefined) | 12 |
| Facility-based screening in Prison | 186 (155-369) | 2 |
| Facility-based screening in school | 341 (118-950) | 4 |
| Facility-based screening in workplace | 580 | 1 |

Abbreviations

NNS, number needed to screen

## Figure A: Weighted mean number needed to screen stratified by active case finding screening location after removing the 9 facility-based screening studies where pre-diagnosis loss to follow-up estimates were available

Legend

Weighted mean NNS (range)

Abbreviations

NNS, number needed to screen
